# The Combination of Enzymes in the Enhancement of Fibre-Enriched Product Quality: Effects of the Interactions of Dietary Fibre, Gluten Proteins, and Starch Granules on Dough Rheological Properties and Bubble Dynamics

**DOI:** 10.3390/foods14223963

**Published:** 2025-11-19

**Authors:** Xiang Zhou, Simiao Wu, Xinyang Sun

**Affiliations:** 1 College of Food Science and Engineering, Collaborative Innovation Center for Modern Grain Circulation and Safety, Nanjing University of Finance and Economics, Nanjing 210023, China; 1220241338@stu.nufe.edu.cn (X.Z.); wusimiao@nufe.edu.cn (S.W.); 2 Department of Food and Human Nutritional Sciences, University of Manitoba, Winnipeg, MB R3T 2N2, Canada

**Keywords:** fibre-enriched product, combined enzymes, biopolymeric interactions, dough rheological properties, bubble dynamics

## Abstract

Due to the presence of insoluble dietary fibre (IDF), DF-enriched products have a lower consumer acceptance compared to those prepared using a regular formulation. The objective of this review was to focus on a comprehensive utilization of enzymes for improving the DF-enriched dough rheology and bubble dynamics via the regulation of intermolecular interactions between DF, starch granules, and gluten proteins. Xylanase was used to promote the interactions between water-extractable arabinoxylan (WEAX) and gluten proteins, leading to a stronger gluten network and dough liquid film around gas bubbles. Cellulase was applied to promote the breakdown of cellulose, mitigating the adverse impacts of IDF on the gluten structure. Glucose oxidase (GOx) was utilized to facilitate disulfide bond (S-S) formation between gluten proteins, thereby enhancing the gluten strength, gas retention capacity, and bubble stability of dough during processing. Amylase incorporation promoted bubble expansion of high-fibre dough. In conclusion, the review established a solid theoretical framework on how an unpredictable evolution for the rheological behaviour and bubble dynamics of dough during processing could be modified via the complicated interactions involving enzymes and biopolymers. This will contribute to a high-quality development for the fibre-enriched product industry, and also a sustainable promotion of regular DF consumption.

## 1. Introduction

Dietary fibre (DF) has been attracting a great amount of attention due to its health benefits, i.e., a regular consumption of DF against the risk of cancer, obesity, and cardiovascular diseases [1]. The incorporation of DF into a dough formulation increased the nutrient contents of the final product [2]. Nonetheless, the consumer acceptance and overall quality of fibre-enriched products remained way less favourable compared to the non-fibre-enriched ones [2]. To stimulate the consumption of fibre-enriched products from a nutritional perspective, substantial effort is required to improve the processing performance and product quality of fibre-enriched dough.

**Figure 1:**
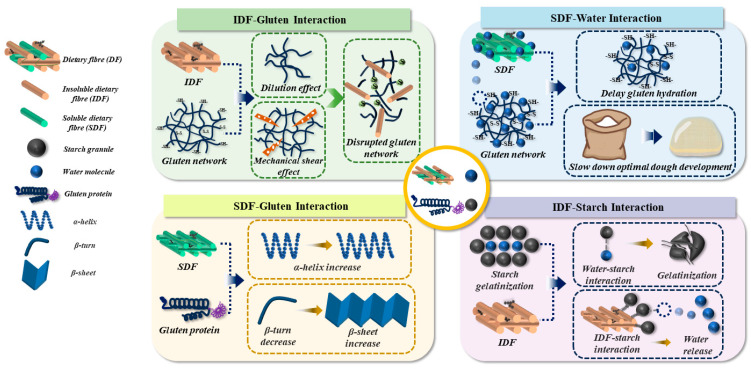
The schematic interpretation of the intermolecular interactions of dietary fibre (DF), gluten protein, starch granules, and water molecules in dough.

The presence of insoluble dietary fibre (IDF) in dough was considered as the primary factor causing quality issues, i.e., coarse crumb structure, firm crumb texture, and small loaf volume, of fibre-enriched products [1]. This can be attributed to the intermolecular interactions between IDF, starch granules, and gluten proteins that cause unanticipated modifications in the physiochemical and microstructural properties of dough [1]. For example, IDF exhibited an interaction with gluten proteins that disrupted the gluten network through the synergistic effects of mechanical shearing and dilution [1]. This led to a negative impact on gas retention capacity and gluten strength of the dough [1]. IDF was also seen to interact with starch granules via non-covalent bonds, i.e., hydrogen bonds and electrostatic interactions, promoting the entanglement and overlap of molecular chains of starch [3]. This inhibits the starch gelatinization during breadmaking, resulting in a reduction in the intestinal digestibility of starch in fibre-enriched products [4]. However, the interactions between soluble dietary fibre (SDF) and gluten proteins in dough were seen to cause the changes in the secondary structure of gluten proteins, i.e., an increase in the content of α-helix and β-sheet, as well as a decrease in the content of β-turn [1]. Contrasted with the gluten proteins, SDF, which have greater amounts of hydroxyl groups in the polymeric chain, was observed to interact with water more efficiently, causing less availability of water molecules for the hydration of gluten protein and thus delaying the optimal dough development time (DDT) [5,6]. As such, the key approach for improving dough processing properties and final product quality is to regulate the intermolecular interactions between DF, gluten proteins, starch granules, and water. This is illustrated in Figure 1.

**Figure 2:**
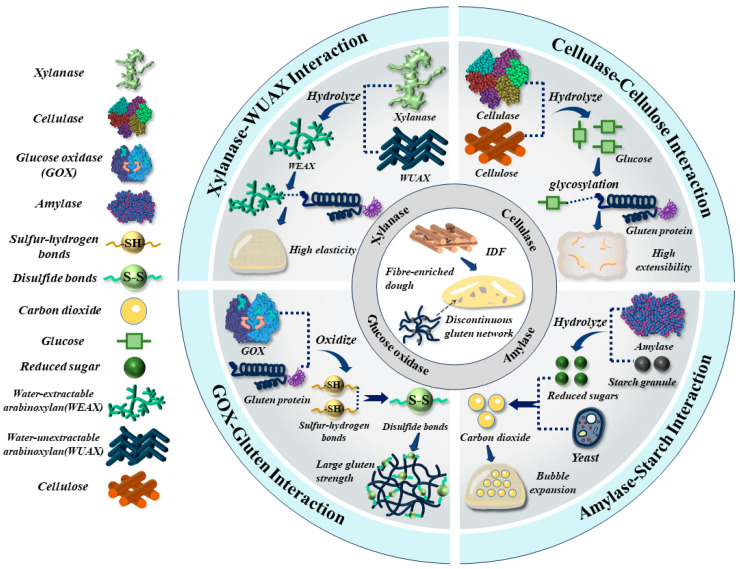
The graphic illustration of the application of combined enzymes in mitigating the negative effects of IDF on the rheological properties and bubble dynamics of fibre-enriched dough.

Enzymes, i.e., cellulase, xylanase, amylase and glucose oxidase (GOx), are known to significantly affect rheological properties and bubble dynamics (defined as a time-dependent evolution for bubble size distribution and stability) of high-fibre dough [7]. This further contributes to an improvement in the overall quality, i.e., finer crumb structure, softer crumb texture, and larger loaf volume [8]. Xylanase was applied to hydrolyze the water-unextractable arabinoxylan (WUAX) into water-extractable arabinoxylan (WEAX), which interacted with gluten proteins to reinforce the gluten structure, and thus enhanced dough elasticity [9]. The interactions between WEAX and gluten proteins also enhanced the stability of the liquid film around gas bubbles, leading to better control of the stabilization and evolution of bubbles in a dough during processing [7,10,11]. Cellulase was seen to hydrolyze the cellulose into cellobiose, glucose, and oligosaccharides [8]. It was observed that the glycosylation between glucose and gluten proteins promoted an unfolding conformation of protein structure, contributing to an enhancement in protein solubility and dough extensibility [12]. GOx incorporation into dough was seen to promote the oxidation of sulfur-hydrogen bonds, and thus the formation of a disulfide bond (S-S) between gluten proteins [13]. This leads to an improvement in the gas retention capacity, bubble stability, and gluten strength of dough throughout processing stages [2]. Amylase incorporation was applied to catalyze the hydrolysis of starch to produce low-molecular-weight polysaccharides and other monosaccharides [14]. This provides a sufficient substrate for yeast to metabolize, thereby generating the CO_2_ and promoting the bubble expansion of dough [14]. This is illustrated in Figure 2.

Previous reviews have demonstrated the use of enzymes in addressing quality issues of fibre-enriched products through the enzymatic modification of the structure and composition of DF in a dough. However, there is a lack of a deeper exploration into the interactions between enzymes, DF, starch granules, and gluten proteins, as well as their effects on dough processing properties. As such, the objective of this review was to demonstrate the effects of enzymes on the intermolecular interactions between DF, starch granules, and gluten proteins, for an effective and efficient regulation of dough rheology and bubble dynamics. The innovation of this work was to establish a solid theoretical foundation on how an unpredictable evolution of the rheological behaviour and bubble dynamics of dough during processing could be modified via the complex interactions involving enzymes and biopolymers. This work also optimizes high-fibre dough formula with a combined utilization of enzymes, achieving the final products with favourable sensory and textural attributes, along with a higher consumer acceptance. This will contribute to high-quality development in the fibre-enriched product industry, and also to a sustainable promotion of regular DF consumption.

## 2. Effects of Combined Enzymes on the Interactions of Dietary Fibre, Gluten Proteins, and Starch Granules

Non-starch polysaccharides (i.e., dietary fibre (DF)) exert a significant effect on dough rheology, i.e., viscoelasticity, extensibility, and water absorption. DF incorporation into a dough system negatively affects the interactions between starch granules and gluten proteins by disrupting the gluten–starch matrix in a dough [15]. As such, enzymes alleviate the negative effects of DF on the gluten structure, thereby inhibiting the DF-induced interruption of the gluten structure in dough. Specifically, xylanases have been applied in a fibre-enriched dough to convert the water-unextractable arabinoxylan (WUAX) to water-extractable arabinoxylan (WEAX), which promotes the transportation of water molecules from gluten proteins to WEAX, resulting in a slower process of gluten hydration [9]. Cellulases are applied to catalyze the hydrolysis of cellulose to alleviate the interference of cellulose [15]. Glucose oxidases act to facilitate disulfide bond (S-S) formation, and also lead to an increase in the gluten macromolecule polymer (GMP) content [13]. This boosts the strength of the gluten network and improves the processing properties of the dough [13]. Amylase incorporation acts to catalyze the hydrolysis of starches in a dough for promoting the yeast-induced fermentation and the Maillard reactions during breadmaking, leading to an improvement in the bread flavour, crumb structure, crust colour, and loaf volume [16]. In addition, a combination of enzymes has also been observed to exert synergistic and antagonistic effects on the processing performance of dough [2]. This is illustrated in Table 1.

### 2.1. Xylanase Effects

Xylan, a highly abundant renewable polysaccharide in nature, consists of monosaccharides interconnected by ester and glycosidic bonds [25]. Whereas xylanase, as a glycoside hydrolyzing enzyme, conducts its catalytic action on the degradation of xylan by cleaving β-1,4-glycosidic bonds to produce xylose and oligosaccharides [23]. The hydrolysis of arabinoxylan (AX) by using xylanases in a whole wheat flour dough increased the water retention capacity of the dough by converting water-unextractable arabinoxylan (WUAX) to water-extractable arabinoxylan (WEAX), and thus mitigating the disruption of insoluble dietary fibre (IDF) on the gluten network [7,26]. This contributes to an improvement in the baking performance of the dough [2]. Xylanases, a member of the hemicellulose family, have been utilized to retard starch retrogradation to a certain extent by controlling the aggregation process of straight-chain starch polysaccharides [27].

Arabinoxylans (AX), as a kind of non-starch polysaccharide, are the main type of hemicellulose that exists in the bran of cereal grains [28]. Depending on their solubility in water, they can be categorized into water-extractable arabinoxylans (WEAX) and water-unextractable arabinoxylans (WUAX) [29]. The AX from wheat bran has a relatively higher water retention capacity due to its deeply branched and porous configuration [30]. Xylanase has been used to hydrolyze the AX by breaking down the xylan backbones, leading to a conversion of WUAX into WEAX that competes with gluten proteins for interaction with water molecules [16]. As such, it slows the process of gluten hydration and increases the viscosity and water retention capacity of dough [17].

Xylanase was used to hydrolyze the WUAX by cleaving β-1,4 glycosidic bonds between xylose residues, causing decreased molecular weight of WUAX and increased water solubility [29]. This causes an enhancement in water retention capacity (WRC) and the development of high-fibre dough [2]. The addition of xylanase in a whole wheat flour dough reduced the WRC of WUAX, caused a release of free water, and, in turn, induced the transportation of water molecules from WUAX to gluten proteins, thereby enhancing the gluten strength and the processing properties of dough [9]. In addition, the hydrolysis of xylan by xylanase converted the WUAX to WEAX, causing a reduction in the WUAX content and mitigating its adverse impacts on the gluten network [2]. In a whole wheat flour dough, the increase in WEAX increased the bubble stability, and, in turn, improved the baking performance of the dough [2]. Xylanase incorporation was applied to convert the WUAX to WEAX and increase the WRC of high-fibre dough, thereby indirectly improving the processing properties of the dough and its product quality [2].

In a whole wheat flour dough, the hydrolysis of arabinoxylans by xylanases resulted in the dough with a lower viscosity, augmenting the mobility of protein fragments and promoting their linkage [8]. This leads to accelerated protein aggregation that is due to the removal of arabinoxylan-induced steric hindrance [7]. The addition of xylanases in a dough also caused the transportation of water molecules from arabinoxylans to gluten proteins [11,26]. This makes the water molecules more available for plasticizing the gluten proteins, promoting the development of the gluten network and thus improving the processing properties of the dough. Therefore, it results in bakery products with a desirable quality, i.e., softer crumb texture and larger loaf volume [6].

Wheat bran, a primary co-product of wheat grain milling, features dietary fibre as its major constituent, which accounts for 35–50% of the total bioactive compounds [5]. In a high-fibre dough system, wheat bran inclusion caused physical interference with the gluten network [31]. This leads to the dilution of gluten proteins and also the competition for water molecules between dietary fibres, starch granules, and gluten proteins [9]. Therefore, xylanases were applied to hydrolyze the non-starch polysaccharides (i.e., dietary fibre) to minimize their damage to the gluten structure [11]. The hydrolysis of arabinoxylan by xylanase in a whole wheat flour dough caused the conversion of WUAX to low-molecular-weight WEAX, altering the fraction of dietary fibre [2]. This mitigates the interruption of the gluten network, which improves the baking performance of the dough [31]. Moreover, xylanase was used to convert the WUAX to WEAX, which induced water molecule transportation from the gluten proteins to WEAX, slowing down the hydration of gluten proteins and thus delaying dough development time [7].

### 2.2. Cellulase Effects

Cellulose has a wide distribution in nature, providing a good source of fibre materials [32]. It exhibits a complex structure composed of covalently linked D-glucose repeating units [32]. The molecular backbone of cellulose is a linear-stiff chain that is linked by the β-D-glucopyranose through 1,4-glycosidic bonds, which is conformed in the shape of a chair [33]. The highly functionalized, linear, stiff-chain cellulose has been characterized by determining its chemical modifying capacity, hydrophilicity, and chirality [33]. Cellulose is capable of dissolving in select solvent systems, i.e., ionic liquids (ILs) and N-methyl morpholine N-oxide (NMMO), but is generally insoluble in most media [34].

Cellulases are a broad class of glycoside hydrolases, synthesized by an array of microorganisms, including fungi, bacteria, and actinomycetes [35]. Cellulases are commonly used to cleave the β-1,4 glycosidic linkages to promote the degradation of celluloses [2]. This is due to the existence of conserved domains and catalytic centres in the cellulase [36]. The cellulase-induced hydrolysis of cellulose is assisted through an acid–base catalytic process that is carried out by two catalytic residues [37]. The hydrolysis is carried out through a retention or inversion of anomeric conformation, depending on the spatial arrangement of residues possessing the specific catalytic activity [37]. Cellulase is categorized as exo-1,4-β-D-glucanase (exoglucanase), endo-1,4-β-D-glucanase (endoglucanase), and β-glucosidase [2]. They act synergistically to promote the hydrolytic degradation of cellulose [38].

Dietary fibre is composed of soluble dietary fibre (SDF) and insoluble dietary fibre (IDF) due to its solubility in water [2]. Wheat bran dietary fibre (WBDF) consists of SDF and IDF, where IDF accounts for 85-93% of WBDF [39]. WBDF incorporation enhances the water retention of dough without increasing its water activity, resulting in an extension in the shelf life of resultant products [2,7]. Moreover, WBDF has been observed to retard starch retrogradation to maintain the product quality over storage time [7].

After the cellulase treatment on WBDF, the content of IDF decreased, mitigating the negative effect of IDF on the continuity of the gluten network, and, in turn, modifying the rheological properties of whole wheat flour dough and the texture of whole wheat breads [40]. The cellulase-induced hydrolysis of cellulose decreased the particle size of cellulose, mitigating the interference of cellulose on the continuity of the gluten network [18]. This prevents the hydration of gluten proteins, decreases the mobility of proteins, inhibits their conformational changes, and, in turn, promotes the aggregation of proteins with a higher molecular weight [41]. Cellulase was seen to hydrolyze the crystalline regions of cellulose, leading to an exposure of more hydroxyl groups than of water within the cellulose structure [19].

The addition of dietary fibre to dough was commonly applied to improve the nutritional values of resultant bread products [2]. This brings some negative effects on the processing properties of dough [42]. These negative effects on whole wheat flour dough are due to the competition for water molecules between insoluble dietary fibre (IDF) and gluten proteins that results in the partial gluten polymer dehydration and subsequent conformational changes [18]. The existence of the IDF also disrupted the continuity of gluten structure from the spatial position [18]. The addition of cellulase facilitated the formation of disulfide bond (S-S) between the two cysteine residues that contributed to the development of stable secondary structures, leading to an enhancement in the degree of protein polymerization that facilitated the formation of gluten network [20]. In addition, the enzymatic breakdown of cellulose by cellulase promoted the conversion of divided protein domains to connected protein strands, facilitating a better gluten network development [18].

### 2.3. Glucose Oxidase Effects

Glucose oxidase (GOx) belongs to a sub-classification of oxidoreductases that is derived from moulds (i.e., *Penicillium idiosyncraticum* and *Aspergillus niger*) and honey [43]. GOx, as a member of the glucose-methanol-choline oxidoreductase (GMC) superfamily, is a flavin adenine dinucleotide (FAD)-dependent oxidoreductase with a folded structure [44]. GOx has been observed to catalyze a transfer of electrons from oxidants to the reductants [43]. Particularly, in the presence of molecular oxygen, GOx catalyzes β-D-glucose to form hydrogen peroxide and D-glucono-δ-lactone via oxidation [44]. The resultant hydrogen peroxide catalyzes the interactions of free sulfhydryl groups, thereby promoting the formation of a disulfide bond (S-S) [13].

In a dough system that is incorporated with insoluble dietary fibre (IDF), the existence of IDF causes the interruption of the gluten network, which negatively affects the optimal development of dough [5]. The addition of GOx modified the interactions between DF and gluten proteins, thereby mitigating the negative effects of IDF on the gluten network [13]. The GOx in the dough was seen to promote the formation of disulfide bonds and an increase in the content of gluten macromolecule polymer (GMP) [2]. This strengthens the continuity of the gluten network and also increases dough stability [45]. In addition, GOx was used to modify the development of gluten matrix in a fibre-enriched dough that increased dough porosity [13].

The incorporation of bran into a dough interrupted the gluten hydration and also yielded a less-polymerized gluten matrix, leading to an elevation of α-helix and β-sheet contents that positively affected dough stability [15]. GOx facilitated the formation of disulfide bonds and induced more stable protein conformations in the gluten matrix [46]. GOx addition contributed to an elevation of GMP content and enhanced gluten protein polymerization [2]. This facilitates the formation of hydrophobic and hydrogen bonds, contributing to the establishment of a stable secondary conformation that is expressed as increased β-sheet and α-helix contents, and thus promoting the development of gluten network [13]. In addition, disulfide bond formation and protein polymerization by GOx inclusion weakened the adverse impacts of IDF on the gluten structure [47]. GOx inclusion into the dough also enhanced dough elasticity and improved final product quality, i.e., finer crumb structure and larger loaf volume [47].

### 2.4. Amylase Effects

Starch, as a storage polysaccharide, is made up of α-D-glucopyranosyl residues linked by α-glycosidic bonds [48]. It includes amylose that is connected by α-1,4 glycosidic bonds, and also amylopectin that is highly branched and mainly connected through α-1,6 glycosidic bonds [49]. Amylases, as an enzyme that hydrolyzes starch, are mainly classified into α-amylase and β-amylase [21]. Within the glycoside hydrolase family, α-amylase is recognized as the most widely applied member for its catalytic functions [50]. It hydrolyzes starch by cleaving α-1,4-D-glucosidic linkages that exist between glucosyl residues and other similar α-glucans in the starch molecules, and hence releases the glucose, maltose, and other low-molecular-weight polysaccharides [21]. As an exohydrolase, β-amylase specifically breaks down α-1,4 glycosidic linkages in D-anhydroglucose monomers at the non-reducing ends of amylose and amylopectin, releasing maltose units from glucan chain termini [49].

Maltogenic amylase (MAA), as a type of α-amylase, is a member of the glycoside hydrolase family 13 that mainly produces the α-maltose [14]. MAA mainly catalyzes the hydrolysis of multiple substrates (i.e., starch, cyclodextrins, and pullulan polysaccharides) and the transglycosylation reactions on the activated glycosyl groups and monosaccharides [51]. MAA has been applied to efficiently hydrolyze cyclodextrins into glucose and maltose, with concurrent transglycosylation of hydrolysate that promotes the synthesis of oligomaltoses [52]. In a whole wheat flour dough system, it was challenging to retard starch retrogradation, while enzymatic treatment was commonly applied to delay or even prevent starch retrogradation [53]. During the starch gelatinization, MAA hydrolyzed the amylopectin to reduce the length of its side chains and produce the by-products, i.e., maltoses, oligosaccharides, and low-molecular-weight dextrins [51]. This inhibits starch recrystallization, promotes the entanglement of protein macromolecules and starch granules, delays starch retrogradation, and, in turn, prolongs product shelf life [53].

The incorporation of amylase into a dough also significantly affects the textural properties of resultant breads [40]. It was observed that amylase addition cleaved α-1,4-glycosidic bonds in starch, generating monosaccharides and low-molecular-weight polysaccharides [2]. This promotes the yeast-induced fermentation of dough and the Maillard reaction during baking, and, in turn, improves the crust colour, bread flavour, and loaf volume of the final product [16]. A large amount of CO_2_ was released in the dough due to the yeast activity, resulting in decreased crumb firmness and increased loaf volume [54]. Amylase facilitated the hydrolysis of amylopectin side chains into short branches, leading to a lower degree of starch granule crystallization and diminished water molecule retention in granule microstructures, as well as subsequent retardation of starch retrogradation [22]. This results in an extension of the shelf life, and also a significant reduction in the crumb firmness of whole wheat breads [40].

### 2.5. Combined Enzyme Effects

Enzymes, as a typical source of dough improvers, are often used in combination to achieve better product quality [24]. A combined use of enzymes mitigated or even inhibited some negative effects of dietary fibre (DF) on dough processing properties [40]. DF, as non-starch polysaccharides, exerted a significant effect on the development of the gluten network in a high-fibre dough [15]. The interactions between DF and gluten proteins were observed to induce a conformational change in the gluten matrix and a disruption of the continuity of the gluten network [2]. This disruption of the continuous gluten network negatively affected the resultant product quality [15]. Currently, enzymatic modification is commonly applied to mitigate the adverse impacts of incorporated DF on dough processing performance [2]. During the breadmaking process, the synergistic action of enzymes was observed to modify the dough rheology, i.e., an enhancement in bubble stability and an improvement in dough extensibility [6].

A synergistic action between glucose oxidase (GOx) and xylanase (XYL) acted to affect gluten structure and composition [23]. When used in combination rather than alone, GOx and XYL in whole wheat flour dough systems significantly reduced free sulfhydryl group levels and enhanced glutenin macropolymer (GMP) accumulation, promoting an enhancement in the strength of the gluten network [23]. In addition, the application of GOx and XYL to a whole wheat flour dough also resulted in decreased β-turn levels and increased β-sheet levels that were considered as the most stable secondary conformation in gluten proteins, thereby boosting dough mechanical properties [47].

The synergistic action of XYL and GOx was also observed to weaken the interactions between WUAX and gluten proteins [46]. The above enzymes hydrolyzed the WUAX into small fragments, facilitating the aggregation of gluten protein and the development of the gluten network, as well as improving the gas retention capacity in the dough [24]. With XYL and GOx incorporated into a whole wheat flour dough system, it caused an increase in the content of WEAX and gluten proteins, whereas GOx catalyzed the crosslinkings of the above biological polymers [46]. This leads to an improvement in the strength of the gluten network, and also an enhancement in the resistance of the dough liquid film to thermal stress [45]. It further causes a retardation of the bubble disproportionation and coalescence, and improves bubble stability in a dough and final product qualities, i.e., finer crumb structure and larger loaf volume [45].

Synergistic utilization of enzymes, i.e., xylanase and cellulase, was applied to a whole wheat flour dough system to hydrolyze the insoluble cellulose and arabinoxylans into various monosaccharides [2]. This promotes the dough fermentation and Maillard reaction, facilitating increased loaf volume, enriched bread flavour, and improved crust colour of final products [55]. In addition, the hydrolysis of these non-starch polysaccharides by xylanase and cellulase in a fibre-enriched dough also promoted the transportation of water molecules from gluten proteins to soluble dietary fibre (SDF), slowing down gluten hydration and thus delaying the dough development time [55]. This results in a modification for dough processing properties, i.e., finer crumb structure and larger specific volume [2]. Moreover, the incorporation of α-amylase and xylanase into a whole wheat flour dough system was observed to enhance dough extensibility, resulting in an improvement in resultant product quality [6].

## 3. Effects of Combined Enzymes on the Rheological Properties and Bubble Dynamics of Fibre-Enriched Dough

The incorporation of wheat bran dietary fibre (WBDF) into a wheat flour dough system has been observed to negatively affect the strength of the gluten network, which causes a reduction in dough extensibility and viscoelasticity [2]. Moreover, insoluble dietary fibre (IDF) inclusion interferes with the continuity of the gluten network, negatively affecting bubble stability and gas retention capacity [2]. This further negatively affects final product quality, i.e., uneven crumb structure and lower loaf volume [56]. The individual and synergistic applications of enzymes, i.e., xylanase, glucose oxidase (GOx), and amylase, act to improve dough rheology and bubble dynamics [40]. This is illustrated in Table 2 and Table 3.

### 3.1. Large-Strain Rheological Properties of Dough

Large-strain measurements are known to characterize dough rheology, i.e., consistency, stability (STA), and development time (DDT), which are related to the resultant product quality [6]. The measurements include the mixograph, farinograph, and mixolab [2]. The mixograph serves as a commonly used mixing device for dough preparation [6]. The mixograph metrics, i.e., peak height, mixing development time, work input, and peak bandwidth, are employed to characterize how ingredients influence dough rheology [6]. The farinograph, a commonly employed device, assesses the mechanical properties of dough through the mixing of flour with sufficient water [58]. During the farinograph test, the measured parameters include the STA, DDT, and mixing tolerance index (MTI), which directly indicate the strength of gluten proteins in a dough [6]. The mixolab determines the thermomechanical properties of dough throughout the mixing process, heating, and cooling [61]. The mixolab parameters include the mechanical weakening, DDT, STA, starch retrogradation, starch gelatinization, and water absorption [2].

The viscoelastic behaviour of gluten proteins has a direct relationship with the processing properties and resultant product quality of dough [58]. It has been observed that the contents of glutenin and gliadin in a dough are directly correlated with the formation and development of the gluten network, which positively affects the elasticity and extensibility of the dough [58]. The addition of GOx to a whole wheat flour dough facilitated the formation of disulfide bonds and induced more stable protein conformations in the gluten matrix, which contributed to an increase in the gluten macromolecule polymer (GMP), i.e., higher-molecular-weight glutenin, and enhanced gluten protein polymerization [2]. This facilitates an enhancement in the strength and stability of the gluten network, directly affecting the dough rheology [2]. Moreover, an increase in the content of HMW glutenin subunits in the dough also led to a higher tolerance of the dough to overmixing and an enhancement in the gluten strength, thereby improving final breads with a larger loaf volume [2].

In a wheat flour dough system, water mixes with wheat flour and thus interacts with gluten proteins to promote the formation of intermolecular hydrogen bonds between gluten proteins, leading to an enhancement in the stability of the gluten network, and in turn has positive effects on the rheological properties, i.e., an enhancement in the elasticity of the dough [58]. The content of water molecules at the mixing stage of the dough affected the hydration of gluten proteins, which caused significant changes in the development time of the dough [6]. In a whole wheat flour dough system, the incorporation of wheat bran dietary fibre (WBDF) competed with gluten proteins for interacting with water molecules, slowing down the hydration of gluten proteins, and thus delaying the optimal development time of the gluten network [15]. The addition of cellulase and xylanase hydrolyzed the insoluble dietary fibre (IDF) into low-molecular-weight soluble dietary fibre (SDF), which caused a transportation of water molecules from gluten proteins to SDF in a fibre-enriched dough [2]. This causes a decrease in the amount of available water molecules for gluten hydration, delaying the optimal development time of gluten proteins [9]. However, with increasing the water content in a dough, the resistance and consistency of the dough decreased, indicating a weakening effect of excessive water on the dough [58].

The individual use of GOx in a whole wheat flour dough facilitated the formation of disulfide bonds and an increase in the content of GMP, which caused an enhancement in the strength of the gluten network [13]. This leads to a reduction in the extensibility and an enhancement in the gluten strength of the dough [57]. However, the incorporation of XYL into a GOx-induced dough converted the WUAX to WEAX, which competed with gluten proteins for interacting with water molecules, causing fewer available water molecules for the gluten hydration of the dough, and thus delaying the optimal development time [57]. Soluble pentosan (i.e., WEAX) addition inhibited the aggregation between gluten proteins, leading to more available gluten proteins for water interaction [57,62]. This causes a reduction in the gluten strength, as well as an enhancement in dough extensibility [57].

### 3.2. Small-Strain Rheological Properties of Dough

The small-strain measurements, i.e., creep-recovery tests and dynamic oscillatory rheometry, have been conducted for determining the fundamental rheological properties of dough at various processing conditions and ingredient [58]. Compared to the large-strain measurements, the small-strain measurements cause non-destructive changes for the dough samples due to a relatively small stress that is applied to the dough [58]. Dynamic oscillatory rheometry is employed to measure the elastic (storage) modulus G′ and viscous (loss) modulus G″ of wheat flour dough as a function of angular frequency in a linear viscoelastic region [2]. It has been shown that dough prepared from wheat cultivars with stronger gluten (i.e., higher gluten strength) has higher values of G′(ω) and G″(ω), along with lower tan δ (G″/G′) [58]. Creep–recovery tests are employed to evaluate the time-dependent strain (γ) and compliance (J) of dough under a constant shear stress σ_0_, presenting results as the creep–recovery strain γ (t) or compliance J (t) = γ (t)/σ_0_ [58]. At both creep and recovery phases, the recovery J_r_(t) and creep J_c_(t) compliances have been used to characterize the viscoelastic characteristics of dough within nonlinear deformation regimes [58].

In a whole wheat flour dough, it was observed that the presence of insoluble dietary fibre (IDF) disrupted the continuity of the gluten structure, which caused a reduction in the gluten strength [2]. The enzymes, i.e., xylanase and cellulase, were used to hydrolyze the IDF into soluble dietary fibre (SDF) that mitigated the IDF-induced disruption of the gluten network [20]. The interactions between SDF, starch granules, and gluten proteins promoted the formation of a continuous film-like matrix that involved intermolecular crosslinkings between the three biopolymers as above [5]. This interaction strengthens gluten structure, consequently positively affecting the extensibility and elasticity of dough, as well as boosting the viscoelastic moduli (G′ and G″) of dough [2]. In addition, the formation of crosslinked biopolymers also enhanced the resistance to deformation and strength of dough, which significantly decreased dough creep strain (γ) from creep–recovery tests [5]. DF, containing abundant hydroxyl groups, was tightly bonded with free water molecules in the dough that caused a movement of water molecules from gluten proteins to DF, weakened the gluten–water interactions and thus increased the gluten strength, thereby enhancing dough mechanical properties and viscoelasticity [63]. Therefore, the DF-induced competition for water molecules into a high-fibre dough also led to a reduction in dough creep strain (γ) [5].

In a whole wheat flour dough system, the individual and combined use of enzymes significantly modifies the small-strain rheological properties of dough, i.e., changes in G′ and G″ values [17]. The addition of xylanase and cellulase in a whole wheat flour dough hydrolyzed the insoluble dietary fibre (IDF), i.e., cellulose and water-unextractable arabinoxylan (WUAX), into soluble dietary fibre (SDF), i.e., water-extractable arabinoxylan (WEAX) and monosaccharides with a lower molecular weight [58]. This promotes a transportation of water molecules from gluten proteins to IDF in the dough, leading to a decrease in the content of water molecules interacting with gluten proteins and thus an increase in the values of G′ and G″ of the dough [58]. Moreover, the addition of GOx in a whole wheat flour dough facilitated the formation of disulfide bonds and also promoted the aggregation of gluten protein, leading to an increase in the gluten strength and the G′ of the dough [13]. However, when GOx and XYL were simultaneously incorporated into the dough, XYL exhibited a prominent effect on the hydrolysis of WUAX [23]. The XYL-induced hydrolysis of WUAX mitigated the negative effect of WUAX on the continuity of the gluten network, thereby boosting gluten strength, as indicated by higher G′ and G″ values [2].

The creep–recovery compliance (J) of the dough demonstrated a relationship with the elasticity and viscosity of the dough [58]. The presence of WUAX in a whole wheat flour dough was seen to interact with gluten proteins through sulfur–hydrogen bonds (-SH), which indirectly inhibited the intermolecular interactions between gluten proteins, causing an adverse impact on disulfide bond (S-S) formation, and thus a decrease in the gluten strength [2]. Xylanase addition in a whole wheat flour dough facilitated the hydrolysis of WUAX, which caused the exposure of -SH on the surface of gluten proteins, thereby accelerating disulfide bond (S-S) formation between gluten polymer chains [2]. This promotes the crosslinking of gluten proteins, thereby elevating gluten strength and resulting in a reduction in the creep compliance (J_c_) of the dough [2].

Gluten–starch dough has been used as an ideal model to investigate the interactions between starch granules, gluten proteins, and other components of dough during processing [64]. At the stage of mixing, gluten proteins were developed into a three-dimensional network, and also interacted with water to proceed the hydration [6]. Starch granules were observed to interact with water and thus start the gelatinization [20]. This starch gel had a strong interaction with gluten proteins due to its function as a filler across the gluten network [20]. However, the incorporation of dietary fibre (DF) in a gluten–starch dough was observed to exert a competition between DF, starch granules, and gluten proteins for water interaction because of the greater water-holding capacity of DF [65]. This causes an unfavourable water molecule redistribution within a gluten–starch dough, thereby resulting in a partial dehydration of gluten proteins and starch granules [65]. This further results in a decrease in dough consistency, and thus negatively affects final product quality, i.e., coarse crumb structure and smaller loaf volume [65]. In addition, the WRC of DF is known as a key determinant shaping the changes in gluten–starch dough rheology [66]. It was also observed that in a gluten–starch dough, the interactions between DF, starch granules, and gluten proteins indirectly slowed down the hydration of gluten proteins and delayed the optimal dough development time [65]. This intervention causes a reduction in the gluten strength and dough elasticity [63]. However, DF particle size exhibited no significant influence on high-fibre dough rheology and microstructure [67].

In a gluten–starch dough with water-unextractable arabinoxylan (WUAX), xylanase and glucose oxidase (GOx) were used to modify dough viscoelasticity and extensibility [23]. Single application of GOx in a gluten–starch dough accelerated disulfide bond (S-S) formation between gluten polymer, resulting in an enhancement in the gluten strength and the viscoelasticity of dough [13]. Xylanase application in a gluten–starch dough catalyzed the hydrolysis of WUAX into low-molecular-weight water-extractable arabinoxylan (WEAX) [8]. Xylanase-mediated hydrolysis of WUAX was seen to cause an improvement in the continuity of the gluten network and an increase in the gluten strength of fibre-enriched dough [23]. Synergistic application of GOx and xylanase in a gluten–starch dough further resulted in a transformation of protein secondary structure from β-turn to β-sheet, thereby enhancing gluten network stability and dough elasticity, as well as favouring an improvement in dough processing performance [23].

### 3.3. Bubble Dynamics in the Dough

In a whole wheat flour dough system, bubble dynamics, i.e., a time-dependent evolution for the entrainment, growth, break-up, disentrainment, disproportionation, and coalescence, have been considered as the primary factors of gas bubble stabilization and retention during processing [56]. Throughout the breadmaking processes, gas bubbles account for roughly 9–20% of total dough volume at the end of mixing, 70–75% of that at the end of proving, and finally 75–85% of that at the end of baking [56]. At the mixing stage of dough, gas bubbles are entrapped in the dough when the surface of the dough approaches each other due to the mechanical action of the mixing instrument, shown as the entrainment of gas bubbles [2]. According to the hypothesis of dough liquid film, many individual smaller-sized bubbles are embedded into the continuous gluten–starch matrix that is considered the liquid film around the bubbles in a dough by the end of mixing [56]. The entrainment of gas bubbles is positively correlated with the number and volume of gas bubbles in the dough [56]. Bubble disentrainment in a dough causes a removal of gas bubbles, reducing the void fraction (VF) of the dough [68]. However, it has been observed that increasing the mixing time results in an increase in the VF of the dough [69]. During the breadmaking processes, bubble disproportionation occurs at the initial phase of dough fermentation [56]. According to Henry’s law, bubble disproportionation was caused by gas diffusion from the smaller-sized bubbles with a higher pressure on their surface to the larger-sized bubbles with a lower pressure on their surface [70]. This results in an increase in larger-sized bubbles, as well as a shrinkage and even disappearance of smaller-sized bubbles in the dough [56].

During proofing and the early stages of baking, due to the yeast activity in the dough, it produces a large amount of CO_2_ in the dough [2]. With the increased amount of CO_2_ and rise in temperature, it promotes the diffusion of CO_2_ from the dough matrix into the bubbles, leading to an expansion in the bubble sizes and also an increase in the VF of the dough [56]. In a typical straight-dough process, punching, that is, the mechanical pressing of dough, was completed after the first stage of fermentation [70]. This causes a further subdivision of bubbles with smaller sizes, promoting an even bubble size distribution (BSD) of dough [70]. During the later stage of proving and baking, the liquid film between adjacent bubbles further thins, resulting in a decrease in the distance between bubbles [56]. This causes a reduction in the electrostatic and steric repulsive forces at bubble-liquid interfaces that drives bubble coalescence within the dough matrix [71].

It was seen that bubble disproportionation and coalescence result in a reduction in the number of bubbles and also a loss of gas in the dough [56]. This negatively affects final product quality, i.e., coarse crumb structure and lower loaf volume [2]. Therefore, to obtain the bread with a large loaf volume and even distribution of cell sizes in the crumb, both disproportionation and coalescence need to be prevented during breadmaking processes [68]. Higher strain hardening at the interface of expanding bubbles in a dough has been observed to enhance the stress around bubbles that have undergone substantial growth [67]. The enhancement results in a reduction in the driving force for gas transportation from smaller- to larger-sized bubbles that mitigates or even inhibits the disproportionation and coalescence, leading to an enhancement in the bubble stability during the dough processing [56]. Moreover, the addition of surface-active agents into the dough has also been observed to reduce the surface tension on the interface between bubbles [6]. This strengthens the dough liquid film around bubbles that increases the bubble stability against coalescence and disproportionation, leading to an improvement in the crumb structure and loaf volume of resultant breads [56].

Bubble stability has been observed to exhibit a positive correlation with gluten network, indicating that stronger doughs have a higher strain hardening during the breadmaking processes [70]. Larger stress has been applied to the dough if a higher strain is caused by the dough deformation [2]. Higher strain hardening of dough favours an enhancement in the bubble stability against coalescence and disproportionation, facilitating uniform bubble expansion throughout breadmaking processes [70]. It has been observed that the addition of xylanase (XYL) and glucose oxidase (GOx) in a high-fibre dough significantly enhances bubble stability of the dough when the temperature rises [47]. This occurs because the addition of GOx in a whole wheat flour dough promotes the formation of disulfide bonds (S-S) and, in turn, the aggregation of gluten proteins, thereby enhancing gluten strength and improving bubble stability [47]. In addition, the XYL incorporation causes a conversion of water-unextractable arabinoxylan (WUAX) to water-extractable arabinoxylan (WEAX) with a lower molecular weight, which inhibits the diffusion of gas bubbles from the inside to the outside of the dough [9]. The inhibition occurs because of the presence of WEAX, which acts as a steric stabilizer in the liquid film surrounding gas bubbles, thereby improving bubble stability within the dough matrix [47].

Bubble size distribution (BSD), that is, the total number of bubbles as a function of their sizes for per unit dough volume, has been found to be positively correlated with final product quality metrics [56]. During breadmaking, bubble growth and expansion cause a reduction in the average diameter of bubbles in a dough [47]. The incorporation of glucose oxidase (GOx) and xylanase into a whole wheat flour dough facilitates the conversion of WUAX to WEAX, and also accelerates disulfide bond (S-S) formation of gluten polymers [13,17]. This promotes the crosslinkings between WEAX and proteins at the liquid film around bubbles, facilitating the formation of stronger liquid films that resist thermal stress [47]. As such, it contributes to an improvement in the bubble dynamics, i.e., lower growth rate, smaller average sizes, and higher stability of gas bubbles in the dough [47].

Amylase addition has been observed to affect dough expansion, and also the crust colour and crumb structure of resultant products [60]. In a whole wheat flour dough system, amylase and xylanase acted to hydrolyze starch granules and non-starch polysaccharides, i.e., pentosans, into low-molecular-weight polysaccharides and monosaccharides [53]. This provides a sufficient substrate for yeast to digest and thus produce the CO_2_, leading to a promotion for the bubble growth and the dough expansion [14]. Furthermore, it has also been found that during the long-term storage of breads, the retrogradation of starch granules results in a reduction in the cell size of resultant products [72]. This negatively affects final product quality, i.e., lower loaf height and volume [56]. The presence of amylase in a dough hydrolyzed the amylopectin into short-chain ones and decreased the crystallization degree of starch granules, and, in turn, delayed starch retrogradation and extended the shelf life of resultant products [60].

The strain hardening index (SHI) of dough is positively correlated with the loaf volume of resultant products [73]. Research indicates that the strain hardening of gluten proteins in a dough facilitates a uniform growth of bubbles, and also retards or even prevents bubble coalescence and disproportionation [56]. In a gluten–starch dough system, the addition of wheat bran dietary fibre (WBDF) causes a thinning effect and even remarkable damage to the gluten structure, thereby reducing the strain hardening of the liquid film [67]. This causes a reduction in bubble stability and a negative effect on the baking performance of gluten–starch dough [67]. The WBDF in a gluten–starch dough interacts with gluten proteins to disrupt the continuity of the gluten network, which causes a decrease in the gas retention capacity of the dough [74]. This inhibits the uniform expansion of bubble sizes throughout the proofing and baking, causing an adverse impact on the overall quality of bakery products, i.e., an uneven distribution of cell sizes in the crumb and smaller loaf volume [75]. In addition, WBDF inclusion into a gluten–starch dough has also been observed to form a physical barrier around the gas bubbles [74]. This negatively affects the uniform growth of bubbles at the stage of proofing and baking [75].

## 4. Effects of Combined Enzymes on the Overall Quality of Fibre-Enriched Products

The incorporation of dietary fibre (DF) in a wheat flour dough system promotes an increase in the content of DF, micronutrients, and other bioactives that contribute to an improvement in the nutritional properties of resultant products [76]. However, the existence of insoluble dietary fibre (IDF) negatively affects both the textural and physicochemical properties of resultant products [5]. Enzymes, i.e., cellulase, amylase, peptidase, and protease, were applied to enhance fibre-enriched product quality [2]. For example, the incorporation of xylanase and cellulase into a whole wheat flour dough was seen to hydrolyze the IDF into SDF and other monosaccharides, mitigating or even inhibiting IDF-induced adverse impacts on dough properties [8]. Cellulase- and xylanase-mediated hydrolysis of IDF resulted in an increase in the amount of reducing sugars that provided sufficient substrate for yeast to digest and thus produce CO_2_, and also promoted the Maillard reaction [59]. This results in expanded bubble sizes and diminished crumb hardness, as well as an enhancement in the crust colour, bread flavour, and consumer acceptance of breads [59]. This is illustrated in Table 4.

### 4.1. Physical and Chemical Properties

At the mixing stage of dough, flour is mixed with sufficient water to form a dough with a continuous gluten network that traps and retains gas bubbles during processing [6]. It has been observed that gas retention capacity and gluten strength are positively correlated with the overall quality, i.e., crumb texture and loaf volume, of resultant breads [2]. The presence of wheat bran dietary fibre (WBDF) in a dough system disrupted the interactions between gluten proteins, leading to a negative effect on the continuity of the gluten network and the gluten strength of the dough, as well as the overall quality of resultant products [6]. Enzyme incorporation mitigated the negative effect of WBDF on the gluten network and gluten–starch matrix, promoting an improvement in the gluten strength and baking performance of fibre-enriched dough that exhibited a correlation with final product quality [8].

It has been seen that in a whole wheat flour dough system, enzyme incorporation results in reduced consistency and enhanced extensibility of the dough [78]. Moreover, amylases were used to hydrolyze the starch granules into low-molecular-weight polysaccharides and monosaccharides that provided a sufficient substrate for yeast fermentation to produce a large amount of CO_2_, leading to a promotion for bubble growth and dough expansion [14]. This further leads to a reduction in the crumb firmness and an improvement in the loaf volume [78]. Moreover, α-amylase, as a kind of endoenzyme, accelerated the breakdown and gelatinization of starch granules in a dough, promoting a reduction in the molecular weight of starch [79]. This causes a negative effect on the starch gel and a reduction in the crumb hardness [79].

The incorporation of xylanases into a whole wheat flour dough system was found to cause an improvement in the structural characteristics of resultant products [2]. Xylanases acted on the backbone of water-unextractable arabinoxylan (WUAX) and then converted the WUAX to water-extractable arabinoxylan (WEAX) and other monosaccharides, i.e., arabinose and xylose [11]. This causes the release of bound water from WUAX and promotes the transportation of water molecules from the WUAX to gluten proteins, facilitating an enhancement in the extensibility of dough [9]. The hydrolysis of WUAX by xylanases mitigated the negative effects on the continuity of the gluten network, facilitating an enhancement in the gluten strength [9]. This improves the gas retention capacity of dough during proving and baking, thereby increasing the specific volume of whole wheat breads [2]. Glucose oxidase (GOx) incorporation into a dough catalyzed the oxidation of glucose into gluconic acids and hydrogen peroxide (H_2_O_2_), indirectly facilitating the covalent crosslinkings between gluten proteins [13]. This enhances the gluten strength of the dough and causes a decrease in the hardness of the resultant products [2].

The addition of proteases and peptidases in a whole wheat flour dough system has been observed to cause remarkable changes in the secondary structure of gluten proteins, which enhances their emulsifying capacity [8]. Protease and peptidase were applied to hydrolyze the gluten proteins into low-molecular-weight peptides and free amino acids with more hydrophilic polypeptides, resulting in the cleavage of peptide bonds, and also an unfolding of gluten structure in a dough [80]. This facilitates the interactions between peptides and lipids that contribute to a fixation of peptide molecules at the oil–water interface, resulting in lowered interfacial tension and boosted emulsifying capacity of gluten proteins [8]. Moreover, the hydrolysis of proteins by proteases and peptidases in a whole wheat flour dough facilitated a further formation of polypeptide chains that favoured bubble entrainment at the stage of mixing, promoting an improvement in the formation of gas bubbles in a dough [8]. The hydrolysis of gluten proteins by the protease in a wheat flour dough also caused an exposure of hydrophobic groups on the gluten surface that enhanced the hydrophobic interactions between gluten proteins, leading to an enhancement in the gluten strength of the dough [81].

During breadmaking processes, enzyme addition at different concentrations (2%, 4%, and 6%) had no significant influence on the pH value of bakery products [40]. At the stage of baking, the water evaporation and chemical processes, i.e., yeast activity, starch gelatinization, and amylase-induced hydrolysis of starch, were observed to cause a decrease in the pH from 6.0 to 5.5 for the resultant breads [82]. Enzyme incorporation, i.e., amylase and xylanase, into a wheat flour dough exerted no significant impact on the water activity of resultant breads [40].

In a gluten–starch dough, the incorporation of soluble dietary fibre (SDF), i.e., inulin (IN) and polydextrose (PD), significantly affected the rheological and microstructural properties of the dough [4]. During the breadmaking processes, the addition of IN and PD, with a higher water retention capacity, contributed to an enhancement in the viscosity of dough, and an increase in the cohesiveness of fibre-enriched products [4]. In addition, it was observed that the existence of IN and PD caused a significant reduction in the water activity of resultant products [83]. SDF in gluten–starch dough was seen to interact with gluten proteins via hydrogen bonds, boosting gluten network strength, and increasing the crumb chewiness and hardness of bakery products [77]. It was also observed that the existence of IN and PD in a gluten–starch dough inhibited the evaporation of water molecules during breadmaking [4]. This favours good control for the growth and expansion of gas bubbles, causing a lower porosity and denser cell structure of bread crumbs [4].

### 4.2. Sensory Evaluation

The sensory properties of bakery products include the flavour, aroma, appearance, crust colour, crumb structure, loaf volume, and consumer acceptability [84]. The addition of wheat bran dietary fibre (WBDF) in a whole wheat flour dough system broke down the fibrils of gluten proteins and disrupted the continuity of the gluten network, which caused a reduction in the gas retention capacity of the dough [63]. This negatively affects the baking performance of the dough, which is associated with the overall quality of final products [2]. Therefore, to obtain the bread with a large loaf volume and even distribution of cell sizes in the crumb, enzymes, i.e., cellulose, xylanase, amylase and glucose oxidase (GOx), were used to mitigate and even prevent the negative effects of WBDF on the gluten network [2]. This leads to an enhancement in the gluten strength, and also an improvement in the baking performance of fibre-enriched dough [13].

The addition of cellulase, xylanase, and amylase in a whole wheat flour dough system hydrolyzed the starches and non-starch polysaccharides, i.e., cellulose and pentosans, into low-molecular-weight polysaccharides and the monosaccharides that increased the content of reducing sugars (RS) [40]. The increased content of RS in a whole wheat flour dough provided sufficient substrate for yeast fermentation, thereby generating CO_2_ and facilitating the Maillard reaction during baking [77]. This leads to larger bubble sizes, softer crumb texture, as well as better crust colour, bread flavour, and consumer acceptability of breads [77]. Moreover, the application of xylanase and cellulase in a whole wheat flour dough was seen to hydrolyze the insoluble dietary fibre (IDF), i.e., arabinoxylans and celluloses, into soluble dietary fibre (SDF), thereby reducing the detrimental impacts of IDF on gluten structure [8]. This enhances the gluten strength that is correlated with the baking performance, ultimately improving the consumer acceptability of fibre-enriched dough [2].

Glucose oxidase (GOx) incorporation in a whole wheat flour dough interacted with soluble pentosans, which increased the consistency of the dough [13]. GOx incorporation into a wheat flour dough system induced the formation of hydrogen peroxide (H_2_O_2_) that catalyzed the interactions between free sulfhydryl groups, and thus promoted the formation of disulfide bonds (S-S) between gluten proteins, leading to an increase in the content of gluten macromolecule polymer (GMP) of dough [13]. This enhanced the gluten structure of the dough and improved the overall quality, i.e., finer crumb structure [2]. Protease was used to break down the gluten network that weakened the gluten structure and thus enhanced dough extensibility [85]. Lipases were observed to interact with the triacylglycerols, phospholipids, and diacylgalactolipids in whole wheat flour dough that produced large amounts of polar lipids [85]. This contributed to an enhancement in the mechanical properties and stability of dough during processing, improving texture properties, i.e., softer crumb texture, of breads [85]. This further improved the consumer acceptance of resultant products [31].

Flavourzyme, as a commercial peptidase from *Aspergillus oryzae*, consists of a variety of exopeptidases and endopeptidase [86]. It has been observed that the incorporation of flavourzyme into a dough promotes the formation of volatile compounds, contributing to an improvement in the sensory properties, i.e., taste and aroma, as well as the consumer acceptability of resultant products [86]. The endopeptidases were used to hydrolyze the polypeptide chains that contributed to the release of free peptides with a lower molecular weight in a dough [80]. The existence of exoproteases in a whole wheat flour dough acted on the end of polypeptide chains that induced the release of free amino acids (FAA), and thus produced the volatile aromatic compounds due to the microbial metabolism during the breadmaking processes [80]. The increased content of FAA and peptides with a low molecular weight in a dough was considered a good source of nitrogen for promoting the growth of yeast, and also enhancing its activity during breadmaking [86]. This leads to the accumulation of organic acids and the emergence of cheesy aroma in baked goods [86]. The existence of FAA and peptides in a dough acted as the aroma precursors for the Maillard reaction, and thus enhanced the attractive sensory attributes, i.e., caramelized aroma, toasted and nutty flavour, for the resultant products, favouring an increase in their consumer acceptability [86].

### 4.3. Nutritional Characterization

A regular intake of dietary fibre (DF) from fibre-enriched products contributes to reducing the health risk of cardiovascular diseases [5]. The presence of insoluble dietary fibre (IDF) negatively affects gluten strength and bubble stability of fibre-enriched dough, thereby impacting bakery product quality [5]. The incorporation of cellulase mitigated and even inhibited the negative effect of IDF on the gluten network, thereby improving the gluten strength and elasticity of the dough [40]. Cellulases were used to hydrolyze the IDF into soluble dietary fibre (SDF) that provided a series of positive physiological effects on the health aspects of human beings, i.e., a reduction in plasma cholesterol level, an attenuation in the glycaemic response, and an improvement in the prebiotic functions [77].

The balanced ratio of SDF and IDF in a whole wheat flour dough is considered a critical factor, causing a lower glycemic index (GI) [87]. GI is defined as the ratio of blood glucose level induced by the intake of the tested food and the control [59]. GI values have been used to indicate the health risk for obesity, diabetes, and cardiovascular diseases [59]. It was observed that the intake of SDF, i.e., polydextrose (PD), from fibre-enriched products enhanced the viscosity of gastrointestinal tract residues, thereby delaying the gastric emptying, retarding the intestinal absorption of starches, and in turn decreasing the postprandial glycaemia and lowering the values of GI [59]. Additionally, the existence of SDF in a whole wheat flour dough system reduced the available water molecules for starch gelatinization because of its higher water retention, slowing down the rate of starch granule swelling in breadmaking [59]. This further inhibits α-amylase-mediated hydrolysis of starch gel, blocking reducing sugar production, and in turn lowering the GI of resultant products [59].

In wheat bran, arabinoxylan (AX) is a predominant type of polysaccharide, accounting for approximately 60–70% of the total dietary fibre (DF) in it [66]. Endoxylanase incorporation into a whole wheat flour dough hydrolyzed the AX into arabinoxylan-oligosaccharides (AXOS) [76]. The consumption of AXOS-enriched products was observed to provide a sufficient amount of the short-chain fatty acid butyrate for human beings [76]. Wheat bran dietary fibre (WBDF), as a prominent source of dietary fibre (DF), was incorporated into a dough to promote an increase in the content of micronutrients and other bioactives, i.e., polyphenols, thereby improving the nutritive properties of the final product [76].

The intake of polyphenols has been proven to prevent several diseases due to the metabolism of gut microbiota, i.e., obesity, diabetes, and autoimmune diseases [83]. It has been observed that the polyphenols block the catalytic sites of enzymes, i.e., amylase and glucosidase, inhibiting their catalytic activities on the hydrolysis of starch and non-starch polysaccharides and slowing down the digestion rate of these polysaccharides [83]. Due to the strong binding capacity, amylose interacts with polyphenols to form complexes that prevent the interaction between amylase and amylose, slowing down the digestion process of amylose [83]. Ferulic acid (FA), as a primary source of polyphenols in wheat bran, has a variety of functions, i.e., lowers the cholesterol level in blood and thus prevents coronary heart disease, showing similar health benefits as those from antioxidants and antithrombotic agents [63]. The incorporation of xylanase, cellulase, and feruloyl esterase into a whole wheat flour dough catalyzed the cleavage of ester bonds linking celluloses, xylans, and FAs, thereby releasing free FAs in a dough [2].

In a gluten–starch dough, the existence of soluble dietary fibre (SDF), i.e., polydextrose (PD) and inulin (IN), exerted a significant reduction in the starch digestibility in bakery goods [4]. It was observed that the digestibility of starches was related to the microstructure and gelatinization of starch granules [4]. The IN and PD were observed to interact with starch granules via non-covalent bonds to modify the microstructure of starch granules [88]. This led to an increase in the starch viscosity that inhibited its movement in the small intestine and lowered the absorption level of glucose [88]. Due to a higher water retention capacity, the IN and PD competed with starch granules for water interaction, which slowed and even inhibited starch gelatinization in a dough during breadmaking, leading to a decrease in the digestibility of starches [83].

## 5. Conclusions

The incorporation of insoluble dietary fibre (IDF) in wheat flour dough contributes to an improvement in the nutritional properties, but causes a negative effect on the overall quality, i.e., smaller loaf volume and coarse crumb structure, of resultant products. To overcome the processing challenges and solve the quality issues of fibre-enriched products, a variety of studies have focused on the utilization of enzymes, i.e., xylanase, cellulase, glucose oxidase (GOx) and amylase, for the optimization of dough formulation. The incorporation of xylanase and cellulase in a dough has been observed to hydrolyze the IDF, mitigate or even inhibit its negative effects on the elasticity and gas retention capacity of fibre-enriched dough. GOx has been used to promote the formation of disulfide bonds (S-S) between gluten proteins, leading to an enhancement in the gluten strength and gas retention capacity of dough. Amylases have been used to hydrolyze the starches and decrease their crystallization degree. This delays the retrogradation of starch granules during the long-term storage of breads.

In conclusion, the individual and combined use of the above enzymes has been proven to significantly modify the rheological properties and bubble dynamics of fibre-enriched dough, which are associated with the overall quality of resultant products. As such, this work fills the research gap from previous studies by providing a theoretical foundation on the accurate selection of suitable enzymes in a fibre-enriched dough formulation, contributing to an improvement in the baking performance of dough. It has also innovatively indicated that the gluten–starch dough can be used as an ideal model to demonstrate the intermolecular interactions between dietary fibre, gluten proteins, and starch granules, which brings a deeper exploration into the mechanisms of the combined enzymes for regulating the dough processing properties and its product quality.

## Figures and Tables

**Table 1:** Effects of combined enzymes on the intermolecular interactions of dietary fibre, gluten proteins, and starch granules.

Enzymes	Intermolecular Interactions	References
Xylanase	Hydrolyze the WUAX to WEAX with a lower molecular weight	[16]
	Promote the interaction between WEAX and gluten proteins	[9]
	Slow down the hydration of gluten proteins and delay the optimal development of dough	[17]
Cellulase	Hydrolyze the cellulose to decrease the content of IDF	[18]
	Contribute to the redistribution of water molecules from the gluten proteins to SDF	[19]
	Promote the formation of secondary structure and disulfide bonds for gluten proteins	[20]
Glucose oxidase	Modify the interactions between DF and gluten proteins	[13]
	Contribute to the formation of disulfide bonds and an increase in the content of gluten macromolecule polymers	[2]
Amylase	Hydrolyze the starches into the polysaccharides with a low molecular weight and monosaccharides	[21]
	Inhibit the recrystallization of starch granules, promote the entanglement of protein macromolecules and starch granules, and delay the retrogradation of starch granules	[22]
Combined enzymes	Decrease the content of free sulfhydryl and promote the interaction between gluten proteins	[23]
	Weaken the interaction between WUAX and gluten protein, hydrolyze the WUAX into fragments	[24]

**Table 2:** Effects of combined enzymes on the rheological properties of fibre-enriched dough.

Enzymes	Flour Type	Rheological Properties	References
Xylanase	Weak cultivar	Increase water absorption (%)	[8]
	Weak cultivar	Enhance elastic modulus G′(ω) and viscous modulus G″(ω) (Pa)	[9]
	Medium cultivar	Decrease dough development time (min)	[57]
	Medium cultivar	Decrease creep-recovery compliance J(t) (Pa^−1^)	[2]
	Strong cultivar	Decrease dough consistency	[2]
	Strong cultivar	Enhance dough stability (min)	[5]
Cellulase	Weak cultivar	Increase water absorption (%)	[2]
	Medium cultivar	Decrease dough consistency	[9]
	Weak cultivar	Decrease dough development time (min)	[58]
	Strong cultivar	Enhance dough stability (min)	[2]
Glucose oxidase	Weak cultivar	Increase dough development time (min)	[13]
	Strong cultivar	Enhance G′(ω) and G″(ω) (Pa)	[2,13]
	Weak cultivar	Increase mixing tolerance index (MTI)	[2]
	Medium cultivar	Enhance dough stability (min)	[13]
	Strong cultivar	Decrease dough resistance to extension (g)	[23]
Combined enzymes	Weak cultivar	Decrease dough development time (min)	[20]
	Medium cultivar	Increase dough extensibility (mm)	[2]
	Strong cultivar	Enhance G′(ω) and G″(ω) (Pa)	[23]
	Weak cultivar	Enhance dough stability (min)	[17]

**Table 3:** Effects of combined enzymes on the bubble dynamics in fibre-enriched dough.

Enzymes	Flour Type	Bubble Dynamics	References
Xylanase	Weak cultivar	Increase bubble sizes	[59]
	Weak cultivar	Improve gas retention	[53]
	Medium cultivar	Enhance bubble stability	[47]
	Strong cultivar	Promote bubble production	[9,47]
Cellulase	Weak cultivar	Increase bubble sizes	[59]
	Strong cultivar	Promote bubble formation	[53]
Amylase	Weak cultivar	Increase bubble sizes	[14,53,59]
	Medium cultivar	Promote bubble formation	[53,60]
Glucose oxidase	Weak cultivar	Improve gas retention	[13,23]
	Strong cultivar	Enhance bubble stability	[23,47]
Combined enzymes	Weak cultivar	Enhance bubble stability	[47]
	Strong cultivar	Decrease bubble sizes	[47]
	Weak cultivar	Promote bubble formation	[53]
	Medium cultivar	Inhibit bubble expansion	[13,47]

**Table 4:** Effects of combined enzymes on the overall quality of fibre-enriched product.

Enzymes	Product Type	Physiochemical Properties	Sensory Properties	Nutritional Properties	References
Xylanase	Noodle	Increase loaf volume Decrease crumb hardness	Aroma, flavour, colour, consumer acceptability	Elevate the levels of total SDF and short-chain fatty acid butyrate	[76]
	Bread	Increase loaf volume Decrease crumb hardness	Aroma, flavour, colour, consumer acceptability	Reduce the starch digestibility Lower the GI value	[59]
Cellulase	Noodle	Increase loaf volume	Springiness, gumminess, chewiness, cohesiveness, crumb fineness	Elevate the levels of total SDF Reduce the starch digestibility Lower the GI value	[40,77]
	Bread	Decrease crumb hardness	Aroma, flavour, colour, consumer acceptability	Elevate the levels of free FAs	[2]
Amylase	Bread	Decrease crumb hardness Increase loaf volume	Aroma, flavour, colour, consumer acceptability, crumb fineness	Extend the shelf life of product	[22]
Glucose oxidase	Bread	Decrease crumb hardness	Springiness, chewiness, cohesiveness		[13]
Combined enzymes	Noodle	Decrease crumb hardness	Aroma, flavour, colour, consumer acceptability	Release the free FAAs Reduce the starch digestibility	[8]
	Bread	Increase loaf volume Increase water activity	Aroma, flavour, colour, consumer acceptability	Elevate the levels of total SDF	[40]

## Data Availability

No new data were created or analyzed in this study. Data sharing is not applicable to this article.

## Abbreviations

DF	dietary fibre
SDF	soluble dietary fibre
IDF	insoluble dietary fibre
WBDF	wheat bran dietary fibre
-SH	sulfur-hydrogen bonds
FAA	free amino acids
AX	arabinoxylan
WUAX	water-unextractable arabinoxylan
WEAX	water-extractable arabinoxylan
VF	void fraction
GOx	glucose oxidase
BSD	bubble size distribution
S-S	disulfide bonds
GMP	gluten macromolecule polymer
WRC	water retention capacity
NMMO	N-methyl morpholine N-oxide
ILs	ionic liquids
GMC	glucose-methanol-choline oxidoreductase
FAD	flavin adenine dinucleotide
MAA	maltogenic amylase
XYL	xylanase
DDT	development time
STA	dough stability
MTI	mixing tolerance index
HMW	high molecular weight
SHI	strain hardening index
H_2_O_2_	hydrogen peroxide
IN	inulin
PD	polydextrose
RS	reducing sugar
GI	glycemic index
AXOS	arabinoxylan-oligosaccharides
FA	ferulic acid
